# Semiconductor Gas Sensors for Detecting Chemical Warfare Agents and Their Simulants

**DOI:** 10.3390/s23063272

**Published:** 2023-03-20

**Authors:** Zygfryd Witkiewicz, Krzysztof Jasek, Michał Grabka

**Affiliations:** Institute of Chemistry, Faculty of Advanced Technologies and Chemistry, Military University of Technology, 00-908 Warsaw, Poland; zygfryd.witkiewicz@wat.edu.pl (Z.W.); krzysztof.jasek@wat.edu.pl (K.J.)

**Keywords:** MOS sensor, chemiresistor, field effect semiconductor sensor, CWA

## Abstract

On-site detection of chemical warfare agents (CWAs) can be performed by various analytical techniques. Devices using well-established techniques such as ion mobility spectrometry, flame photometry, infrared and Raman spectroscopy or mass spectrometry (usually combined with gas chromatography) are quite complex and expensive to purchase and operate. For this reason, other solutions based on analytical techniques well suited to portable devices are still being sought. Analyzers based on simple semiconductor sensors may be a potential alternative to the currently used CWA field detectors. In sensors of this type, the conductivity of the semiconductor layer changes upon interaction with the analyte. Metal oxides (both in the form of polycrystalline powders and various nanostructures), organic semiconductors, carbon nanostructures, silicon and various composites that are a combination of these materials are used as a semiconductor material. The selectivity of a single oxide sensor can be adjusted to specific analytes within certain limits by using the appropriate semiconductor material and sensitizers. This review presents the current state of knowledge and achievements in the field of semiconductor sensors for CWA detection. The article describes the principles of operation of semiconductor sensors, discusses individual solutions used for CWA detection present in the scientific literature and makes a critical comparison of them. The prospects for the development and practical application of this analytical technique in CWA field analysis are also discussed.

## 1. Introduction

Despite the signing and ratifying of the CWC [1] by almost all countries in the world [2], the use of chemical weapons must still be considered both on the battlefield and in terrorist attacks. Due to their high toxicity, chemical warfare agents (CWAs) should be detected at concentrations lower than their toxic threshold concentrations [3] and in a short time, preferably in the place where contamination occurs. By performing on-site analysis, the time from sample collection to result is greatly reduced, and the risk of sample contamination during transport is minimized. At the same time, analyses of this type are characterized by much lower accuracy and a lower level of unambiguity than laboratory analyses. Nevertheless, the accuracy of field analyses is often sufficient for first responders, and the results of such analyses enable them to make quick decisions. For this reason, techniques for the detection of chemical warfare agents that can be used in portable analyzers are the subject of intensive research by numerous scientific groups.

So far, the dominant analytical techniques in this field include ion mobility spectrometry-IMS [4,5], flame photometry-FP [6], Fourier transform infrared spectroscopy-FT-IR, Raman spectroscopy and mass spectrometry-MS [7], as well as some combined techniques such as GC-MS [8,9]. Devices based on these techniques allow the detection of CWAs at low concentrations and in a short time. At the same time, these devices, in particular mass spectrometers and GC-MS, are quite complicated and expensive to purchase and operate.

In addition to the above-mentioned analytical techniques, devices using simple and undemanding chemical sensors, such as electrochemical sensors [10], acoustic wave sensors [11], colorimetric and fluorescent sensors [12,13], as well as semiconductors sensors, can be used to detect CWAs in field conditions. Among the above-mentioned, especially the semiconductor sensors attract a lot of interest from numerous research teams.

The group of semiconductor sensors includes a number of devices using various transducers (resistors, semiconductor diodes, metal-insulator-semiconductor-MIS capacitors, MIS field-effect transistors-MISFET, etc.), the common feature of which is the semiconductor material used as a receptor. To put it simply, the semiconductor materials used can be divided into two groups: oxide semiconductors and non-oxide semiconductors [14]. Oxide semiconductor sensors that historically appeared first in gas detection [15] mostly use materials based on SnO_2_, ZnO or WO_3_. Despite many years since their first application, these sensors are still intensively developed and used to detect various gases [16,17,18]. Oxide semiconductors are resistant to high temperatures and other environmental factors, which is why they can be used, e.g., in resistive sensors operating in the air at temperatures of several hundred degrees. On the other hand, non-oxide semiconductors (silicon, conductive organic polymers, carbon nanostructures, etc.) can be used in resistive sensors operating at room temperature and in MISFET sensors and MIS capacitors.

The review of scientific works in the field of the use of semiconductor sensors for the detection of chemical warfare agents allows us to see both the material/basic research (development of innovative semiconductor materials, sensitizing dopants, structural forms, research on the mechanisms of phenomena occurring in a semiconductor material) and construction/application works (testing of transducers, sensor manufacturing technologies, sensor arrays). In these works, instead of actual chemical warfare agents, their simulants are predominantly used, i.e., substances with similar physicochemical properties, but with much lower toxicity.

In the available literature, you can also find several review articles published in recent years on semiconductor sensors for detecting CWAs [19,20,21,22]. All of the above works concern only resistive sensors, mostly with metal oxide semiconductors (MOS sensors). To our knowledge, however, there is no systematic review summarizing the achievements in the field of CWA detection using the entire group of devices classified as semiconductor sensors, including sensors using other types of transducers.

In this review, we present the current state of knowledge and achievements in the field of various semiconductor sensors used to detect chemical warfare agents and their simulants. The work divides the sensors according to the criterion of the semiconductor material used, additionally distinguishing the type of transducer used. The review was mainly based on reports published in the scientific literature, but commercially available solutions are also included. The article also discusses the advantages and limitations of individual types of sensors and the prospects for their development.

## 2. The Characteristics of Semiconductor Sensors

According to the IUPAC definition [23], a chemical sensor consists of a receptor and a transducer. The task of the receptor is to transform chemical information into a form of energy that can be measured by a transducer and transformed into a useful analytical signal. In the case of semiconductor sensors, the semiconductor material acts as a receptor. In the most commonly used semiconductor sensor setup (resistive sensor), this material is also a transducer.

The construction of a semiconductor sensor is determined by the type of transducer used. Based on this criterion, sensors can generally be divided into resistive and field effect sensors. In the case of resistive sensors, a semiconductor resistor serves as a transducer, while in the case of field effect sensors, the transducer is a Schottky diode, FET or MIS capacitor.

### 2.1. The Principle of Operation of a Resistive Sensor

Due to the fact that the dominant group of semiconductor resistive sensors is MOS sensors, the principle of operation of resistive sensors will be discussed briefly on their example (in addition, detailed descriptions of the principle of operation can be found in the works: [14,24,25,26,27]). In this case, the metal oxide semiconductor acts as both a receptor and a transducer. The semiconductor material forming the active layer is usually polycrystalline with present grain boundaries. The change in the resistance of the semiconductor layer (which actually forms the semiconductor resistor) results from the change in the bulk and surface resistance of the grains, the change in contact resistance at the grain interface and the contact resistance between the grains and the electrodes [25]. The processes leading to signal generation differ depending on whether the measured compound is an oxidizing or reducing gas. Examples of oxidizing gases are NO_2_ and O_3_, while reducing gases include, e.g., all CWAs and their simulants. From the point of view of the subject of this work, the principle of operation of MOS sensors during the detection of reducing gases, which is presented below, is particularly important.

In clean air, on the surface of oxide semiconductor grains at a temperature of 100–300 °C [28], oxygen sorption takes place, leading to the formation of anions Oˉ or O_2_ˉ. O_2_ˉ is formed mainly in the presence of very little moisture, while in typical measurement conditions, the Oˉ form dominates [14]. Oˉ ions are formed according to the following equation:

(1) $$O_{2}+2e^{-}\overset{k_{1}}{\to }{2O}^{-},$$

**Figure 1:**
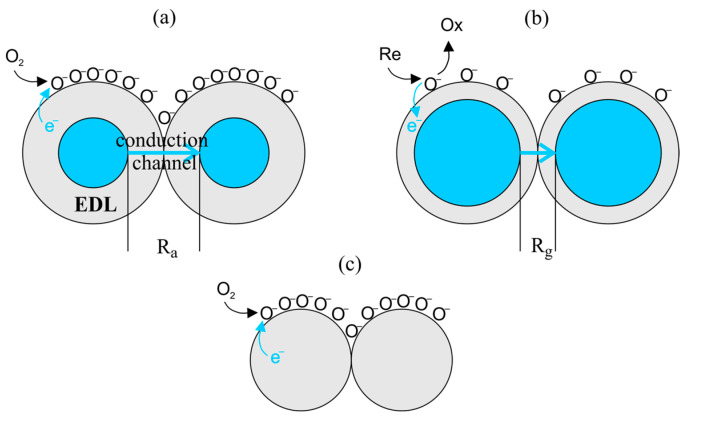
Electron depletion of the grains in n-type semiconductor: (**a**) formation of the electron depleted layer-EDL as a result of O_2_ ionosorption in clean air, the conductivity of the layer is limited by the grain surface concentration of electrons; (**b**) the reducing gas molecule (Re) reacts with the adsorbed oxygen ion forming the oxidized form (Ox) and releasing electrons to the semiconductor-EDL decreases its depth, causing a drop in resistance; (**c**) volume depletion for low-volume grains.

In the case of an n-type semiconductor (e.g., SnO_2_, ZnO), oxygen ionosorption is accompanied by the depletion of electrons near the surface of the grain (regional depletion) and the formation of the so-called electron depletion layer-EDL. As a result, the resistance of the semiconductor will increase. In a p-type semiconductor (e.g., CuO, Mn_3_O_4_), a hole accumulation layer-HAL will be created by removing electrons, and the electrical resistance of the semiconductor will decrease. If the grains have a sufficiently small diameter, the EDL or HAL areas may extend to their entire volume (volume depletion, volume accumulation). The regional and volume depletion situations in an n-type semiconductor are shown in Figure 1.

The surface concentration of electrons (or holes) determines the contact resistance of the grains, i.e., the sensor signal. As a result, in clean air, a certain resistance of the sensor is established, called the base resistance R_a_. When molecules of reducing gas appear in the vicinity of the sensor, they oxidize according to the equation:

(2) $$Re+O^{-}\overset{k_{2}}{\to }Ox+H_{2}O+{CO}_{2}+e^{-}$$

where Re and Ox are the reduced and oxidized forms of the reducing gas, respectively. In the general case, not all oxidized forms are always formed, e.g., for simple gases, such as H_2_ and CO, only H_2_O or CO_2_, respectively, are created. The above reaction is accompanied by the release of electrons from oxygen anions. Depending on the semiconductor material used, the released electrons affect the resistance of the sensor differently. In the case of n-type semiconductors, there will be an increase in the concentration of majority carriers and a decrease in the resistance of the sensor. For p-type semiconductors, the released electrons will recombine with holes, reducing their concentration and, as a result, increasing the resistance of the sensor. In the equilibrium state, both reactions (1) and (2) proceed at the same rate and the resistance of the sensor reaches the value R_g_.

In the case of volume depletion, when a change in resistance under the influence of the reducing gas is significant (R_a_ ≫ R_g_), the change in resistance can be described by the relation [14]:

(3) $$\frac{R_{g}}{R_{a}}=\sqrt{\frac{S(k_{2}/k_{-1})}{{rN}_{D}}P_{M}}=a\sqrt{P_{M}}$$

where S is the grain shape factor; k_2_ and k_−1_ are, respectively, the rate constants of reaction (2) and the inverse reaction (1); r is the grain radius; N_D_, donor density of the semiconductor; and P_M_, the partial pressure of the gas. Thus, the sensitivity (proportional constant a) increases with decreasing grain size and carrier density. However, a decrease in grain size is accompanied by an increase in the response time of the sensor resulting from slower diffusion of gas into the porous layer and a slower rate of removal of oxidation reaction products. Relationship (3) is a particular case of the following power law:

(4) $$\frac{R_{g}}{R_{a}}={aP_{M}}^{c}$$

which works well for many simple systems. For gases, such as H_2_ and CO_2_, as well as O_2_ and O_3_, the constant c ≈ 1/2, while for NO_2_ c ≈ 1 [14]. In the case of organic compounds, many reactions with oxygen ions occur; therefore, these relationships are more complex.

In the literature, the relative response of the sensor for a specific concentration of vapors is usually given (at the same time, the form expressed as a percentage is also common):

(5) $$R=\frac{R_{a}-R_{g}}{R_{g}}$$

**Figure 2:**
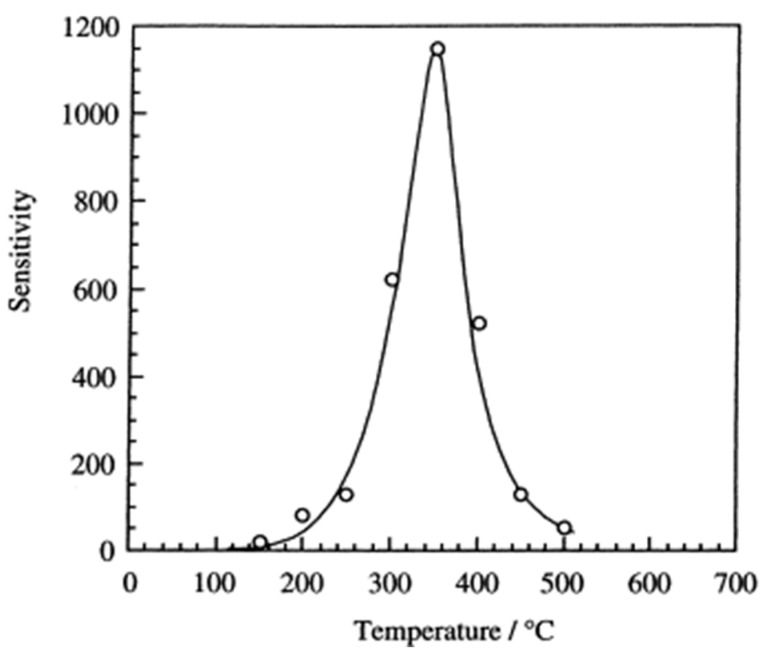
Temperature dependence of sensor sensitivity for a fixed hydrogen concentration of 800 ppm. The peak of the signal is around 350 °C [29]. Reprinted with permission from Ref. [29]. Copyright 2001 Elsevier.

The sensitivity of oxide gas sensors to reducing gases strongly depends on temperature, which results from the overlapping of several processes. As the temperature increases, the reaction rate between the analyte and the adsorbed oxygen (2) increases exponentially, and the gas diffusion coefficient into the porous semiconductor layer increases sublinearly. At lower temperatures, the limiting factor for the signal is the reaction rate of the gas with oxygen. However, at high temperatures, a sharp decrease in the sensitivity of the device is observed, resulting from a decrease in gas concentration in the reaction area. This occurs as a result of gas diffusion into the porous layer and local reduction of gas concentration under the surface of this layer. The effect of this is a non-linear dependence of the sensor response in the temperature domain with a clearly present maximum [29,30]. An exemplary dependence of the change of the sensor’s relative signal with the operating temperature is shown in Figure 2.

**Figure 3:**
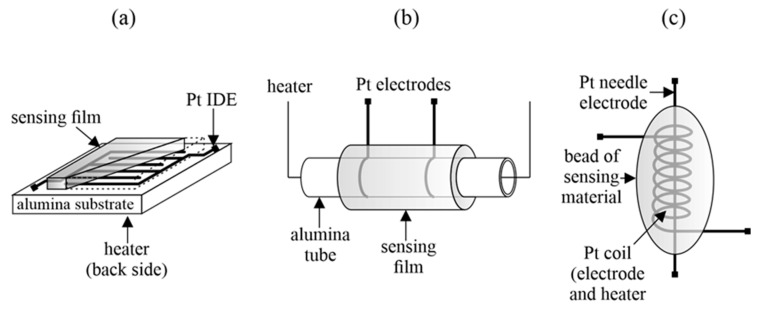
Some designs of resistive semiconductor sensors: (**a**) planar sensor with interdigitated electrodes, (**b**) coated alumina tube, (**c**) small bead with single coil and needle electrode.

A special feature of MOS sensors is that the detection of gases in the air usually takes place at high temperatures (200–500 °C). Therefore, sensors of this type are equipped with a heater that ensures the appropriate operating temperature. A typical sensor consists of a substrate, a heater, a semiconductor resistor and electrodes attached to it for measuring resistance (e.g., interdigitated electrodes-IDE). Figure 3 shows the most common practical arrangements of resistive semiconductor sensors.

In resistive semiconductor sensors, oxide and non-oxide semiconductor materials are used. Sensitizers (often also called dopants), i.e., small amounts of foreign materials, are often added to the base semiconductors, which improve the sensitivity, selectivity, dynamic properties and change the optimal operating temperature of the sensors. For this purpose, metal nanoparticles are commonly used, e.g., Pt, Au or metal oxides (PdO, Ag_2_O, Co_3_O_4_, Fe_2_O_3_, Cr_2_O_3_) as well as semiconductor metal oxides (CuO, CaO). If the role of the sensitizer is limited to the catalysis of the analyte reaction without changing the redox state of the sensitizer, we are talking about chemical sensitization (e.g., Pt particles catalyzing the oxidation reaction of the reducing gas). In this case, the sensitization is due to an increase in the reaction rate of the analyte. The sensitizer can also react with the analyte by changing its redox state. In this case, a change in the form of the sensitizer may cause a change in the resistance of the semiconductor layer by electronic interaction with the semiconductor (e.g., PdO + H_2_ → Pd + H_2_O, Pd + 1/2O_2_ → PdO). In such a situation, we are talking about electronic sensitization [14].

Another sensitivity enhancement mechanism (also classified as electronic sensitization [25]) is based on the fact that the sensitizer inclusion forms semiconductor–semiconductor or metal–semiconductor junctions with the base semiconductor. Thanks to this, the separation of charge carriers is improved and, as a result, the modulation of the sensor response in a wider range is possible [31]. Moreover, sensitizers usually increase the specific surface area of the semiconductor material (they are often characterized by significant porosity). This results in an increase in the number of oxygen vacancies on the surface of the semiconductor and, thus, also in the number of preferred sorption sites for analytes [32].

No redox reactions (either I or II) of the analyte occur in resistive sensors with non-oxide semiconductors. In this case, only the amount of charge carriers in the conduction band changes due to the sorption (often only physical sorption) of the detected molecules on the surface of the semiconductor material. Examples include semiconducting polymers and some types of carbon nanotube sensors.

### 2.2. The Principle of Operation of a Field Effect Semiconductor Sensors

**Figure 4:**
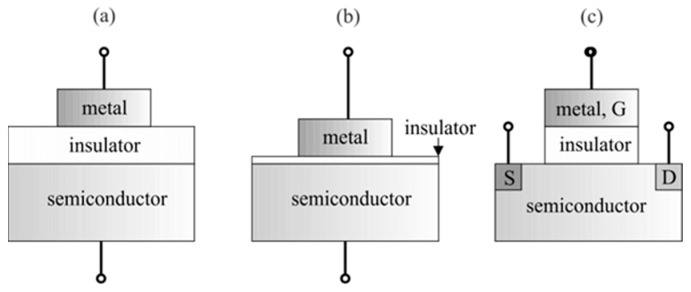
Standard setups of field effect semiconductor gas sensors: (**a**) MIS capacitor, (**b**) Schottky diode, (**c**) MISFET.

Field effect sensors use three different configurations, as shown in Figure 4: MIS capacitors, Schottky diodes and MISFET. In all cases, there is a metal electrode and a semiconductor substrate (usually intrinsic or doped Si) separated by an insulating layer. The role of the receptor is played by a metallic electrode or an additional semiconductor layer placed nearby and in contact with the metal electrode(s). All configurations shown in Figure 4 use the phenomena occurring at the metal–semiconductor interface.

**Figure 5:**
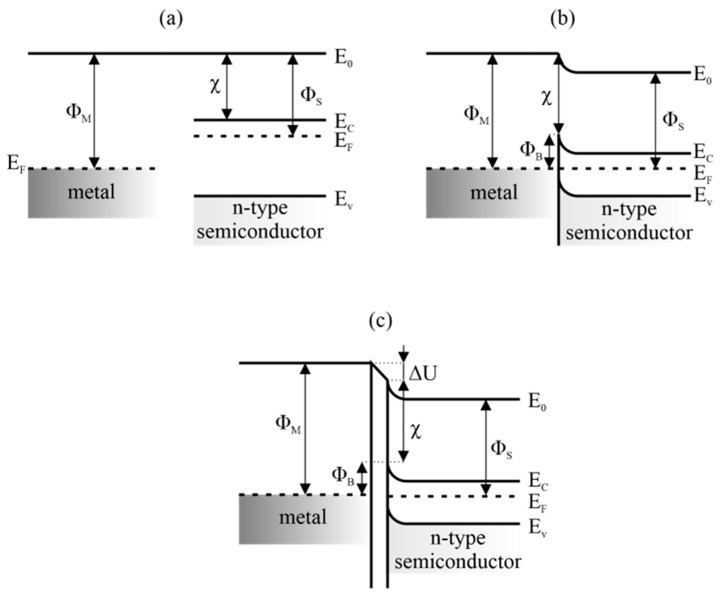
Energy bands of (**a**) isolated metal and n-type semiconductor, (**b**) ideal metal–semiconductor junction, (**c**) model of real junction with narrow gap between metal and semiconductor. E_0_ means the vacuum level or the free-electron energy; Φ_M_ and Φ_S_, metal and semiconductor work function, respectively; χ-electron affinity of semiconductor; Φ_B_, the Schottky barrier; E_F_, the Fermi energy (represents the highest occupied electron energy state at T = 0 K); ΔU, potential difference derived from gas adsorption in the gap between metal and semiconductor.

**Figure 6:**
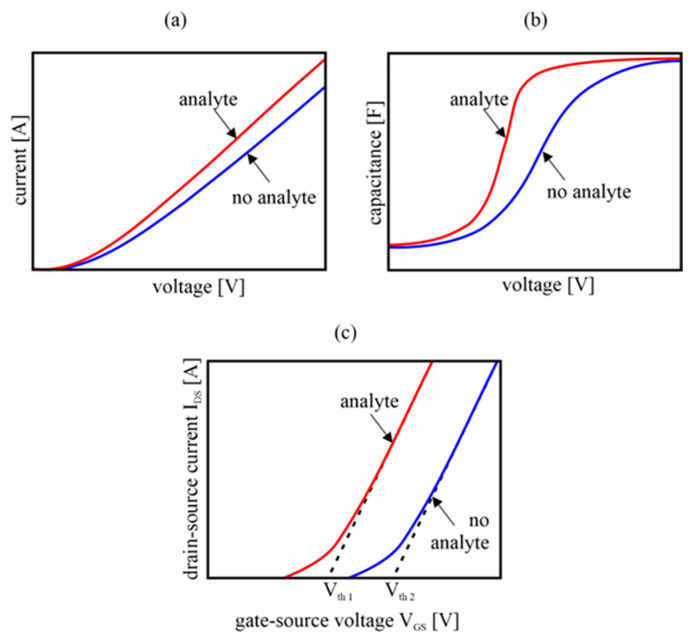
Characteristics of field-effect sensors: (**a**) current–voltage for Schottky diode; (**b**) capacitance–voltage of MIS capacitor; (**c**) drain current–gate voltage for MISFET.

Figure 5a shows the energy bands of the metal and the n-type semiconductor in a situation where both materials are distant from each other. It is worth noting here the different Fermi levels in these materials. After establishing intimate contact between the two materials, the energy bands of the semiconductor bend and a Schottky barrier is created, which modulates the flow of charges through the junction. The Φ_B_ value is equal to the difference between the metal–vacuum Φ_M_ work function and semiconductor–electron affinity χ (Figure 5b). When the role of the receptor is played by a metal electrode, under the influence of the detected gas, the energy bands of the metal shift and the height of the potential barrier in the junction changes. This is observed in the form of a change in the current–voltage characteristics of the diode (Figure 6a).

In the real junction between the rough metal and the porous semiconductor, there are gaps where adsorption of vapors and gases can take place. There, a potential difference ΔU arises, the sign and value of which depend on the charge and the method of polarization of the adsorbate (Figure 5c). From the electrical point of view, such a gap acts as a capacitor whose charge and polarization depend on the concentration and properties of the adsorbed vapors [33]. It is then possible to observe the influence of the tested gas on the capacitive–voltage characteristics of such an MIS capacitor (Figure 6b).

In the FET, the voltage applied between the gate and source (V_GS_) controls the drain-source current (I_DS_). The current starts to flow when the threshold voltage (V_th_) is exceeded and is proportional to (V_GS_−V_th_)^2^, as shown in Figure 6c. Replacing the insulator under the gate with a semiconductor or adding such a layer causes the transistor to react to a change in the gaseous environment, which is observed as a change in the I_DS_ (V_GS_) characteristics, and especially the threshold voltage V_th_ (Figure 6c). Depending on the construction of such a sensor, we can distinguish:

(1) Solid electrolyte–gate FET, where electrochemical half-cells are formed at the point of contact between metal, solid electrolyte and gas.
(2) Oxide semiconductor–gate FET, in which the contact potential between the gate metal and the oxide crystals plays a decisive role.
(3) Dielectric material–gate FET. When a porous dielectric capable of adsorbing vapors and gases is placed under the gate metal, a capacitor is formed whose capacity depends on the gaseous environment. Highly polar vapors change the dielectric constant of the capacitor, which results in a change in the electric field and a shift in the threshold voltage V_th_.

## 3. MOS Sensors

### 3.1. Chemical Reactions of CWAs and Their Simulants Used in MOS Sensors

The available literature on MOS sensors is dominated by works describing the detection of simulants of nerve and blister CWAs. At the same time, there are some papers on the detection of simulants of blood and choking agents. In addition, individual articles describing the detection of actual CWAs can be found [34,35].

**Figure 7:**
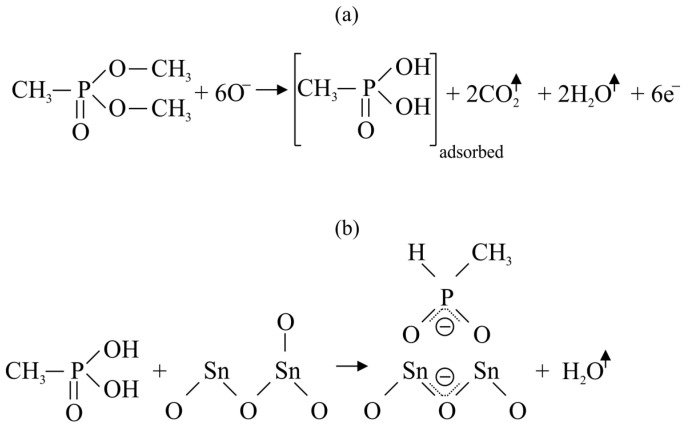
Oxidation of DMMP molecule on the SnO_2_: (**a**) reaction between SnO_2_ and DMMP; (**b**) reaction between methylphosphonic acid and SnO_2_ resulting in adsorption of phosphorus compounds on the oxide surface.

Most of the studies on the detection of nerve CWAs have been conducted with the use of dimethyl methylphosphonate (DMMP), which is a commonly used sarin simulant. The mechanism of the DMMP reaction on the surface of a semiconductor sensor has been studied many times and is well described in the literature [36,37,38,39]. Figure 7 shows the reaction of DMMP with oxygen anions taking place on the surface of SnO_2_ according to a generally accepted mechanism [37].

At a temperature ranging from 300 to 600 °C, DMMP reacts with Oˉ ions, which leads to the release of electrons into the semiconductor and changes in the resistance of the sensor. In the case of DMMP detection, the phenomenon of sensor poisoning is often observed, manifested by prolonged signal recovery and even a permanent decrease in sensor sensitivity. This phenomenon is explained by the fact that the oxidized form of DMMP (methylphosphonic acid) is permanently adsorbed on the surface of the semiconductor (in this case, SnO_2_ (Figure 7b), but the poisoning effect is also observed for ZnO [40], Mn_3_O_4_ [41] and many other materials). As a result, the active surface of the semiconductor available for oxygen ionosorption and reaction with DMMP decreases. The problem of sensor poisoning also occurs with other organophosphate simulants, such as diethyl methylphosphonate (DEMP) [42].

In the case of blister agent detection, numerous tests were carried out with the simulants of sulfur mustard-2-chloroethyl ethyl sulfide (2-CEES) and with the simulant of nitrogen mustard-di(propylene glycol)monomethyl ether (DPGME). There is also work describing research with actual sulfur mustard [34]. In addition, some papers contain studies on the detection of blood and choking agent simulants (primarily dichloromethane-DCM—a simulant of phosgene, and acetonitrile-ACN—a simulant of hydrogen cyanide).

The mechanisms underlying the detection of all these compounds in MOS sensors are very similar. In each case, the analyte is oxidized with the formation of simple gaseous products such as CO_2_, H_2_O, NO_2_, Cl_2_ or SO_2_, which do not cause contamination of the sensor surface (the exception is the detection of DCM, which significantly poisons the SnO_2_ sensor [43,44] and dichloropentane (DCP) [34]). An example of this type of reaction is the oxidation of 2-CEES on the surface of CdSnO_3_ [28]:

(6) $$2\text{-}CEES\to {ClCH_{2}CH_{2}}^{••}SCH_{2}{CH}_{3}$$

(7) $$2\;CH_{3}{CH}_{2}Cl+8\;O^{-}\to 2\;CO_{2}+{Cl}_{2}+4\;H_{2}O+8\;e^{-}$$

(8) $$2\;CH_{3}{CH}_{2}S+13\;O^{-}\to 2\;SO_{2}+2\;CO_{2}+5\;H_{2}O+13\;e^{-}$$

At 300 °C, the 2-CEES molecule breaks down into radicals on the surface of the semiconductor. These radicals then react with adsorbed oxygen ions to release electrons. In this case, the sensor poisoning effect was not observed [28,45,46]. It is also worth noting that in the available literature, there are papers that present in detail the mechanism of ACN detection [47,48,49].

Historically, the first paper in which MOS sensors (SnO_2_, ZnO) were used to detect vapors of organophosphorus compounds was published in 1993 [50]. Over the years, intensive research in this field has resulted in a number of significant achievements, including improvements in the sensitivity, selectivity and stability of sensors. The review of the subjectively most important achievements in the field of MOS sensors in CWAs detection was based on the criterion of the semiconductor material used, separating materials based on SnO_2_, ZnO and other semiconductor oxides.

### 3.2. Sensors Based on SnO_2_

The first generation of semiconductor materials used in the detection of chemical warfare agents and their simulants were polycrystalline (also nanocrystalline) powders, mainly SnO_2_. These materials were usually used in the form of thick layers (films) applied to the sensor surface by screen printing or in the form of pressed disks [43,44,51,52,53,54,55]. In addition to sensors with thick layers (typically 1 to 300 μm thick), sensors with polycrystalline films classified in the literature as thin layers (up to about 1 μm thick) were also used. Thin layers were most often produced by the rheotaxial growth and thermal oxidation (RGTO) method [56,57].

Paper [43] describes the results of testing both undoped and doped SnO_2_ thick film sensors used for DMMP, DPGME, ACN and DCM detection. In this study, SnO_2_ sensors differing in grain size (40 and 15 nm) and applied dopants (Co_3_O_4_, Nb_2_O_5_, MoO_3_, NiO, Sb_2_O_3_) were used. The sensors were operated at 350 °C and exposed to a gas stream with concentrations of simulants ranging from 0.02 to 0.8 ppm. The presented results showed the influence of the grain size, porosity of the semiconductor layer and the type of dopant used on the sensitivity, selectivity and dynamic parameters of the sensors. It was found that dopants significantly affect the sensitivity of the sensor. An example may be a sensor with SnO_2_ doped with NiO, for which the response to DPGME, DMMP, DCM vapors increased by 24, 38 and 1300%, respectively, compared to the undoped sensor. Significant observations also concerned the recovery time of the sensors. In the case of DMMP and DCM detection using undoped SnO_2_ sensors, prolonged recovery times and a decrease in sensor sensitivity were observed. These phenomena were attributed to the poisoning of the sensor surface with the products of analyte oxidation. The magnitude of the poisoning effect was significantly reduced in the case of the MoO_3_-doped sensor.

The issue of optimizing the content and composition of dopants towards eliminating the poisoning effect was developed by the same research team in subsequent works [44,51]. In work [51], a sensor equipped with a thick SnO_2_ layer doped simultaneously with NiO, MoO_3_ and Sb_2_O_3_ was developed, which reportedly showed no poisoning effect during DMMP detection. The influence of individual dopants on the properties of the sensor was empirically identified: the addition of NiO improved the signal amplitude, while MoO_3_ and Sb_2_O_3_ shortened the sensor recovery time. Similarly, work [44] describes a completely reversible SnO_2_ sensor for detecting DCM. In this case, the participation of NiO in the improvement of the response to DCM and MoO_3_ and in the reduction of recovery time was also found.

Further studies, aimed at improving the dynamic parameters of SnO_2_ sensors during DMMP detection are described in [58]. The authors of the cited paper manufactured sensors with thick polycrystalline films made of undoped and doped SnO_2_ nanoparticles. SnO_2_ nanoparticles were obtained by the hydrothermal method and had a diameter of approximately 10 nm. Ni, Sb and Nb were used as dopants. As a reference, the sensor with a commercially available SnO_2_ nanopowder (with an estimated grain size of several tens of nanometers) was used. The results revealed that the sensors with synthesized, undoped SnO_2_ had a much higher sensitivity to DMMP than the sensor with commercial powder (72 vs. 3.1, respectively, for a DMMP concentration of 5 ppm). The increase in sensitivity was attributed to the larger specific surface area of the nanoparticles. At the same time, the sensors with synthesized SnO_2_ returned to equilibrium much slower after switching off the DMMP. Doping with metals significantly shortened the response and recovery times. Particularly good results were achieved for sensors doped with 5% wt. Ni. As reported by the authors, this sensor was characterized by a complete signal recovery in less than 10 min after the DMMP was turned off. At the same time, doping SnO_2_ with Ni significantly reduced the sensitivity of the sensor.

In [52], thick-film SnO_2_ sensors were also studied. In the cited publication, they were used to detect CWAs simulants such as DPGME, DMMP, DCM and ACN. The research was aimed at investigating the influence of the porosity of the semiconductor layer on the sensitivity toward individual analytes. For this purpose, sensors with four different SnO_2_ materials, characterized by different specific surface areas and pore size distribution, were prepared. The results showed that the sensitivity of the sensor is influenced not only by the total specific surface area of the semiconductor layer, but also by the pore size distribution of the layer. For this reason, the sensitivity towards individual analytes differing in mass and molar volume does not increase monotonically with the increase in specific surface area. Research showed that sensors with larger pore sizes were more sensitive to larger molecules (DMMP and DCP). At the same time, materials with a higher specific surface area, but available mainly in micropores, showed lower sensitivity towards these substances.

Sensors with a porous SnO_2_ film were also investigated in [35]. In this case, a sensor with an ethanol-aged, nanoporous thin film of undoped SnO_2_ was presented, which was used to detect real sarin gas (GB). The use of controlled ethanol aging (exposure to 100 ppm ethanol vapor in air at 300 °C for 12 h) significantly changed the performance of the sensor during GB detection. The sensor at a temperature of 300 °C showed unusual behavior for a sensor with an n-type semiconductor in the presence of reducing GB. As a result of the sorption of this gas, the resistance increased as in the case of oxidizing gases. At the same time, the effect was not observed with DMMP. Additionally, at 400 °C, the anomalous behavior towards GB was no longer observed. The authors explain this phenomenon by the adsorption of -CH_x_ groups on the SnO_2_ surface as a result of previous exposure to ethanol. During the detection of GB, the fluorine atom present in GB captures electrons from the -CH_x_ groups and, thus, also from the semiconductor layer, causing an oxidative response of the sensor (increase in resistance). Parallel to this process, GB is oxidized by interaction with Oˉ, and electrons are released into the semiconductor. However, the result of these processes is an increase in the resistance of the sensor. As research has shown, the sensor can detect GB at a very low concentration of 6 ppb and distinguish GB from DMMP.

Noteworthy is also the interesting design solution of the sensor used in [35], which allows for a significant reduction in electricity consumption. The device was made with MEMS technology. Its active part was made of a SiO_2_/Si_3_N_4_ in the form of a square-shaped membrane with a side of 150 μm and a thickness of 1 μm. The membrane, which was integrated with the heater and platinum IDEs, was suspended over the bulk silicon slice via four slender cantilever beams. A film with SnO_2_ was then applied to the membrane surface by the sacrificial template method. In the case of this method, the semiconductor layer is produced using a template (e.g., polystyrene), thanks to which it was possible to deposit a highly ordered monolayer of SnO_2_ nanospheres from a liquid solution (the template itself is burned away in the process of annealing the film). The produced sensor was characterized by a significantly reduced power consumption (25 mW at 350 °C compared to typical 100–300 mW [59]). A detailed description of the manufactured device is included in [60].

A very problematic issue in the case of MOS sensors is the relatively low selectivity resulting from the fact that their principle of operation is based on rather non-specific oxidation and reduction reactions taking place on the grain surface. One of the ways to improve selectivity is the use of appropriately selected dopants. In the case of sensors with SnO_2_ as the basic semiconductor material, the following dopants were most often used: Pt, Pd [47,53], ZnO, ZrO_2_, Al_2_O_3_, In_2_O_3_ [53], CuO, Sm_2_O_3_ [54], Co_3_O_4_, Nb_2_O_5_, MoO_3_, NiO, Sb_2_O_3_ [43]. In most of the published works, the selection of dopants was carried out empirically rather than on the basis of general principles resulting from the mechanisms of the influence of individual dopants on the sensor’s selectivity. One such work is publication [53], which describes a series of thick-film sensors based on SnO_2_ doped with different contents of metals (Pt, Pd) or metal oxides (Al_2_O_3_, In_2_O_3_, ZnO, ZrO_2_). Semiconductor materials were prepared by methods such as impregnation (Pt, Pd), physical milling (Al_2_O_3_, In_2_O_3_) or co-precipitation (ZnO, ZrO_2_). Sensors prepared by screen printing were tested in the presence of DMMP, DPGME, DCM and ACN. As the research showed, the addition of sensitizers significantly influenced the sensitivity of the sensors to individual substances. From among the prepared sensors, four characterized by good sensitivity and stability were selected and used in a sensor array. The analysis of the four-dimensional signal was based on the principal components analysis. As the results showed, the array successfully distinguished vapors of individual simulants.

Another work aimed at improving the selectivity of MOS sensors is [55]. The paper presents an array consisting of 15 commercial MOS sensors (manufactured by Figaro [59]) with polycrystalline SnO_2_ thick films. In the manufacturer’s catalog, individual sensors are described as intended for detecting various organic solvents and gases, but not as targeting CWAs. The intention of the authors was, therefore, to create an array of sensors for CWA detection that meets the definition of a dual-use device. The ability to selectively detect CWAs was to be ensured by using a relatively large number of different MOS sensors and using statistical analysis to interpret the multidimensional signal of the array. In the work, measurements were carried out with nerve CWA simulants such as diethyl diethyl cyanophosphate (DCNP), diisopropyl fluoride, chlorophosphonate and their derivatives, as well as some interferents. As the results showed, the array distinguished individual organophosphorus analytes. In addition, on the basis of the signal, a quantitative analysis was possible (calibration was performed against DCNP only, limit of detection (LOD) of 5 ppm was obtained). Despite the fact that the authors of the study found that the array may be suitable for detecting actual CWA in the field, the LOD values obtained for DCNP significantly exceed the immediate dangers to life or health (IDLH) values of nerve agents [61]. Noteworthy, however, is the good selectivity of the array.

The use of polycrystalline sensor layers, especially those made of coarse grains, is associated with certain limitations. Larger grains are characterized by a lower surface to volume ratio, which limits the active surface on which redox reactions with analyte molecules can take place. Moreover, in the case of such grains, the depletion in charge carriers usually concerns only the surface layer of the grain (regional depletion), and not its entire volume. This results in less change in the conductivity of the layer due to depletion (Figure 1). For this reason, numerous research works focus on the use of semiconductor materials in the form of nanostructures such as: nanostrips, nanowires or nanotubes [56,57,62,63,64]. In the case of nanostructures, it is possible to achieve lateral dimensions comparable to the depth of the charge carrier depletion area. Such materials will, therefore, show volume depletion, and thus, the processes occurring on their surface will strongly affect the conductivity of the semiconductor film. In addition, the peculiar structure of the nanomaterials is characterized by a larger specific surface area. It is also worth noting that the use of single (or parallel) monocrystalline nanostrips or nanowires opens up new perspectives in the field of MOS sensors, consisting of the production of sensors without grain boundaries. Sensors of this type could be characterized by a number of desirable features, such as the lack of long-term drift associated with the reorganization of grain boundaries (grain coarsening) under the influence of high temperatures [65] or reducing the poisoning effect [64].

The work [56] presents a comparative study of MOS sensors equipped with SnO_2_ and In_2_O_3_ nanowires and sensors equipped with traditional polycrystalline oxide layers. Semiconductor nanowires were fabricated using the vapor–liquid–solid (VLS) method. In this method, semiconductor powders are thermally evaporated and then absorbed to a supersaturated concentration in droplets of a catalytic liquid platinum alloy deposited on a sensor substrate. By maintaining appropriate thermodynamic conditions, the growth of single-crystalline nanowires is obtained in accordance with a specific crystallographic direction at the substrate–liquid alloy interface. In the cited paper, sensor responses to vapors of DMMP, DPGME, ACN and other substances (NH_3_, acetone, ethanol and CO) were tested. Research has revealed a higher sensitivity of sensors with nanowires than with traditional films to DMMP and DPGME vapors (allowing their detection at concentrations close to 100 ppb). At the same time, lower sensitivity to other gases, such as NH_3_ and CO, was observed. For all sensors, a strong signal drift was observed, indicating the occurrence of the poisoning effect. The sensor responses were also statistically analyzed to determine their suitability to the sensor array. The authors found that due to significant differences in sensitivity to individual substances, it is advisable to use both sensors equipped with nanowires and traditional polycrystalline films in the array.

A continuation of the above research was presented in [57]. In this case, sensors with nanowires and polycrystalline SnO_2_ films were also compared. The research focused on the qualitative and quantitative description of the sensor poisoning effect by phosphorus compounds during DMMP detection. Research includes testing sensor response to ethanol (25 ppm) and DMMP (0.2 ppm) vapors. The sensors were exposed alternately to these two gases to examine the degree of signal reduction from ethanol under the influence of DMMP poisoning. Studies have shown that the poisoning effect occurs on a similar scale for both types of semiconductor layers. The authors found that the drift of the sensor response in the short term is mainly caused by the contamination of the surface with phosphorus compounds. After a longer period of time without exposure to DMMP (in this work, it was 6 days at 400–500 °C), however, these compounds desorb, and full recovery of the sensor response was possible.

The use of sensors with SnO_2_ nanowires for the detection of DMMP and ACN has also been described in [63]. The study deepened the analysis of sensor poisoning under the influence of DMMP. Research has confirmed that initially, the sensitivity of the sensor decreases rapidly under the influence of repeated exposures to this gas. However, it was found that after long-term exposure (when the product of concentration and time reached approximately 800 ppm·min), the sensor’s response to DMMP stabilized, although the sensitivity was reduced by about 20 times. The effect of sensor poisoning on the detection of ethanol vapors (alternating exposure of the sensor to ethanol and DMMP vapors) was also studied. In this case, it was noted that despite the stabilization of the response to DMMP, subsequent exposures to this gas reduced the response to ethanol, which indicated progressive poisoning. Reduction of sensitivity to ethanol, and probably also to other reducing gases, may have an impact when the sensor is used in an array to distinguish CWAs from interfering gases.

Interesting research results are also presented in [64], which describes a DMMP sensor using a single, monocrystalline, undoped SnO_2_ nanobelt. The aim of the authors was to create a sensor free of grain boundaries. Tests conducted at 500 °C showed that the sensor relative signal reached 5 and 3% at 78 and 53 ppb of DMMP, respectively. Additionally, the authors did not observe the poisoning effect commonly seen with polycrystalline sensors. Noteworthy is the interesting design solution of the sensor setup. To ensure good thermal insulation, a system consisting of a single SnO_2_ nanobelt and a heater (which was also a temperature sensor) was suspended in gas on silicon nitride fibers. Thanks to this, it was possible to significantly reduce the energy consumption of the sensor.

In the recently published work [66], a sensor with oxygen vacancy-enriched SnO_2_ decorated with Au nanoparticles was presented. Modification of commercial SnO_2_ consisted of reaction with (CH_3_)_2_SnCl_2_ and then calcination in air atmosphere. As a result, a significant increase in the concentration of oxygen vacancies on the semiconductor surface was obtained (increase of about 30%; the SnO_2_ modification process was described in detail in [67]). Au nanoparticles in the amount of 5% wt. Au were then deposited on the modified SnO_2_ layer using the in situ reduction method (using HAuCl_4_ aqueous solutions). The sensor prepared in this way was used to detect DMMP. The sensor obtained a relative signal of about 19% at a DMMP concentration of 240 ppb at 320 °C. The detection limit was estimated at 4.8 ppb. The sensor was also characterized by above-average dynamic parameters with response and complete recovery times of 26 and 32 s, respectively. Such good sensor properties were attributed by the authors of the cited paper to the total effect of three mechanisms: increasing the number of oxygen vacancies by modifying the surface with (CH_3_)SnCl_2_, creating metal-semiconductor junctions and thus better modulating the conductivity of the layer and the catalytic effect of Au nanoparticles. The sensor testing results presented in the cited paper are among the best obtained so far for SnO_2_ sensors. It is noteworthy that, in this case, at the optimum operating temperature (320 °C), no poisoning was found, and the sensor was completely reversible.

### 3.3. Sensors Based on ZnO

ZnO is also often used in MOS sensors to detect CWA simulants [40,46,68,69,70]. The advantages of this material include non-toxicity, abundance in the environment and stability. Similar to SnO_2_, ZnO is an n-type semiconductor.

In [71], a sensor with nanocrystalline ZnO powder modified with platinum (0.12% at.) was described. The sensor material was synthesized using the ultrasonic atomization technique and then applied to the substrates by screen printing to form thick films. Platinum was introduced by dipping pure ZnO films into an aqueous solution of chloroplatinic acid and annealing at a high temperature (500 °C). Sensors prepared in this way were tested for DMMP, 2-CEES and 2-chloroethyl phenyl sulfide (CEPS, sulfur mustard simulant). Studies have shown that the sensor with the optimal content of Pt (0.12% at.) was very sensitive and selective towards DMMP (the response to 2 ppm DMMP in 300 °C was about 9 and 26 times higher, respectively, than to 2-CEES and CEPS. Moreover, the sensor showed very good dynamic properties with DMMP response and recovery times of 15 and 45 s, respectively. There was also no sensor poisoning observed with alternating exposure to 2 ppm DMMP.

The work [68] presents the sensor with a polycrystalline ZnO film. Undoped nanoparticles were obtained by the hydrothermal method and had diameters in the range of 30–50 nm. The sensor was used for the detection of 2-CEES and allowed to obtain a relative signal of 15 for a 2-CEES concentration of 1 ppm (estimated LOD of this compound was 0.2 ppm).

In subsequent work published by the same scientific group [46], it was demonstrated that a significant improvement in the sensitivity of ZnO sensors is possible by appropriate doping of the semiconductor material. In the work, sensors doped with the following metals were tested: Al, Co, Cu and Mn. As research has shown, doping with Al significantly improves the sensitivity towards 2-CEES. For the sensor with optimal Al content (1% at.) at 500 °C and for an analyte concentration of 20 ppm, a relative signal of 954.2 was obtained (which is approximately 10 times more than for undoped sensor). In addition, the doped sensor was highly selective toward 2-CEES (the sensor responses obtained for 10 ppm NH_3_, CO and NO were 0.73, 0.14 and 0.11, respectively). As stated by the authors, Al doping increases the concentration of oxygen vacancies on the ZnO surface. As a reason for observing a strong amplification only in the case of Al, the authors indicate a different electron configuration of this element with three valence electrons (in contrast to other used metals). Nevertheless, the work does not provide a detailed explanation and deeper analysis of this issue.

The issue of Al-doped ZnO was continued in [69]. This paper describes a miniature GC system with an MOS detector for the detection of 2-CEES in mixtures. The MOS sensor was equipped with a film of 5 nm ZnO Quantum Dots doped with 1% at Al. The chromatograph used a 5 cm (0.15 cm diameter) packed column with Carbowax 20 M. The column was kept at 30 °C during analysis. Atmospheric air was used as the carrier gas, which was sucked into the system by means of a built-in pump. The sensor acted as a detector at a temperature of 430 °C. The detectability of 2-CEES was 0.5 ppm. Under optimal operating conditions, the retention time of 2-CEED was 150 s. The use of the column enabled the complete separation of CO, NO and NH_3_, which thus did not interfere with the detection of 2-CEES. The intention of the authors was to significantly improve the selectivity of MOS sensors through the use of a combined technique. As the results of the analyses showed, the device was indeed characterized by above-average selectivity, at the cost of a significant complexity of construction. It is also worth paying attention to the parameters of the sensor produced as a detector in the GC system. The sensor was characterized by much higher sensitivity than the previously described solutions. In the case of 20 ppm 2-CEES at 450 °C, a relative signal of 5395 was obtained. The outstanding sensitivity was attributed to the effect of the size of the synthesized nanoparticles (under analogous conditions, a sensor with larger nanoparticles of doped ZnO achieved a signal of 673).

A significant achievement in the field of semiconductor sensors for DMMP detection is described in [70]. The authors fabricated a sensor with a polycrystalline ZnO (average grain diameter of 25 nm) doped with 1% wt. Al. The nanoparticles were produced by the hydrothermal method. Tests have shown that the sensor was characterized by high sensitivity toward DMMP and low LOD. At the optimal operating temperature (350 °C), a relative signal of 4347 was obtained for the DMMP concentration of 10 ppm. At the same time, the estimated LOD of this substance was 100 ppb. The dynamic properties of this sensor are also noteworthy. With a response time of 2 s and a full recovery time of 96 s, it is probably the fastest MOS DMMP sensor reported in the literature. The sensor was also characterized by high selectivity to DMMP. The outstanding properties of the sensor reported in the cited paper were attributed by the authors to an increase in the concentration of oxygen vacancies in doped ZnO. Al^3+^ ions replace Zn^2+^ ions in the crystal lattice of the semiconductor. Due to the smaller ionic size, Al dopants can generate more oxygen vacancies and act as preferential adsorption sites for DMMP. 

One of the ways to improve the sensitivity of MOS sensors is the use of dopants that form semiconductor junctions or metal–semiconductor junctions with the base semiconductor. When two different semiconductors come into intimate contact, due to the differences in the Fermi levels of both materials, energy bands will bend at the contact surface and a potential barrier (Schottky barrier) will be created. Changes in the concentration of charge carriers caused by the redox reaction, taking place on the surface of the semiconductor, affect the height of the created barrier and, thus, also the conductivity of the material. Changes in the conductivity of the layer in the case of an n-p heterojunction are proportional to the height of the potential barrier as follows [31]:

(9) $$\Delta R\sim exp\{-e\Delta \Phi_{B}/k_{B}T\}$$

where: k_B_—Boltzmann constant, ΔΦ_B_—change in height of Schottky barrier, T—temperature.

Particularly interesting results for a system of this type have been described in [40]. In this case, a material based on ZnO nanostructures (called by authors “micron-scale ZnO flowers”) decorated with CuO nanoparticles was presented. In the cited paper, the responses of sensors with CuO/ZnO nanostructures and pristine ZnO nanostructures to DMMP were compared. During the measurements, it was revealed that the CuO/ZnO sensor is characterized by a much higher sensitivity to DMMP than the ZnO sensor and good selectivity towards this substance (the value of the relative signal for 10 ppm DMMP at 350 °C was 626.21, and for the same concentration of NH_3_, CO, NO and NO_2_ values of relative signals did not exceed 50). In addition, the response time of the CuO/ZnO sensor to DMMP was almost 13 times shorter than that of the ZnO sensor (26.2 and 330 s, respectively). The studies have shown that the addition of CuO causes a significant increase in sensor resistance in clean air, which may be caused by the expansion of the EDL by the formation of p-n junctions. At the same time, during exposure to DMMP, the width of the depletion area is reduced, and the resistance decreases to a value close to the value achieved by a sensor with pristine ZnO under the same conditions. As a result, the modulation of the sensor signal is four times wider than in the case of a sensor with pristine ZnO. A detailed analysis of the results contained in the cited publication also allows us to draw conclusions about the short-term repeatability of the sensors. It is clear that the sensors show a decrease in the sensitivity to DMMP during subsequent, repeated exposure cycles. This phenomenon has already been mentioned for SnO_2_ sensors and results from the poisoning of the sensor surface with phosphorus compounds.

### 3.4. Sensors Based on Other Semiconductor Oxides

In addition to SnO_2_ and ZnO-based materials, other oxides were also used in MOS sensors for the detection of CWAs and their simulants. These include materials such as: WO_3_, In_2_O_3_, CuO [34,56,72], MnO_2_, Mn_3_O_4_ [41,73,74,75] or CdSnO_3_ [28,46].

The work [34] presents thick film sensors with undoped WO_3_ and In_2_O_3_ used for the detection of actual CWAs and their simulants. The list of analytes included sarin, soman (GD), sulfur mustard (HD) and simulants (DMMP, DCP—dichloropentane). Both tested sensors showed high sensitivity to CWAs and estimated GB and HD detection limits of 10 ppb at 400 °C. In addition, the sensors were found to be much more sensitive to live agents than to their simulants. An interesting observation was the trend of increasing the sensitivity of the WO_3_ sensor in the group of organophosphorus compounds: DMMP-GB-GD. This phenomenon was attributed to the increase in the number of methyl groups in the analyte molecule. During adsorption of the analyte, these groups are oxidized and constitute a source of additional electrons. As in the case of DMMP for GB and GD, the sensors showed poisoning.

In [28], sensors with thin films of polycrystalline CdSnO_3_ and Pt-doped CdSnO_3_ are described. Several sensors with different thicknesses of semiconductor films were manufactured and tested. As determined by X-ray diffraction, CdSnO_3_ grains were characterized by an average diameter of less than 5 nm. The films were deposited using the ultrasonic spray pyrolysis technique. The manufactured sensors were used to detect sulfur mustard simulants: 2-CEES and CEPS, as well as a sarin simulant-DMMP. The study revealed that both undoped and platinum-doped sensors were highly sensitive and selective towards 2-CEES. In the case of the undoped sensor (with a film thickness of 1.87 μm) at 350 °C, the signals for 4 ppm 2-CEES, CEPS and DMMP were: 12.05, 2.04 and 2.14, respectively. Under the same conditions at 250 °C, the signals for the Pt-doped sensor (1% vol., film thickness 1.14 μm) reached 58.63, 6 and 3.85. As the results showed, Pt doping significantly increased the sensitivity to 2-CEES and lowered the optimal operating temperature of the sensor from 300 to 250 °C. In addition, as the authors indicate, the dynamic parameters of the sensor have improved.

Further work on CdSnO_3_ sensors focused on Ru doping [45]. In this case, as before, the films were produced using the ultrasonic spray pyrolysis technique. Several sensors differing in the percentage content of ruthenium and the thickness of the polycrystalline films were manufactured and tested in the work. 2-CEES, CEPS and DMMP were used in the study. The best results were obtained for a sensor with a Ru content of 1.85% wt. and a film thickness of 365 nm. This sensor was characterized by a relative response to 2-CEES, CEPS and DMMP of 62.12, 7.23 and 4.9, respectively, at 350 °C. By comparing these results to those previously obtained for undoped CdSnO_3_ and platinum-doped CdSnO_3_ [28], it can be concluded that metal-doped sensors are characterized by similar sensitivity and selectivity towards 2-CEES. In the case of the Ru-CdSnO_3_ sensor, a much shorter response and recovery time was obtained (for 4 ppm 2-CEES: 5 and 185 s, respectively). In the case of the Pt-CdSnO_3_ sensor, it was 30 and 300 s, respectively. It is worth noting, however, that these values were obtained at the optimal operating temperatures of the sensors, for Ru-CdSnO_3_: 350 °C and for Pt-CdSnO_3_: 250 °C. Due to the much lower operating temperature, which is reflected in the electrical power consumption and long-term stability, the Pt-CdSnO_3_ sensor is also worthy of attention.

Recently, Mn_3_O_4_ and MnO_2_ sensors have also attracted a lot of interest [41,73,75]. The paper [41] describes sensors with undoped Mn_3_O_4_ layers manufactured by the chemical vapor deposition (CVD) method. Deposition of the layers was carried out in an oxygen atmosphere in the presence of water vapor and in dry oxygen, which resulted in obtaining films with different surface morphology and grain sizes (80 nm and elongated grains 110 × 270 nm, respectively). The sensors were tested in the presence of CH_3_CN (hydrogen cyanide simulant) and DMMP. The results showed that the sensor manufactured in a humid oxygen atmosphere was more sensitive to CH_3_CN than the second sensor. At the same time, both sensors have similar sensitivity to DMMP. Unfortunately, in both cases, significant sensor poisoning was observed during the detection of DMMP. Mn_3_O_4_, as a p-type semiconductor, decreased its conductivity under the influence of the detected reducing gases (CH_3_CN, DMMP). The response time for CH_3_CN at 300 °C at a concentration of 2 ppm was 1 min, and the recovery time was 8 min. DMMP at 200 °C at a concentration of 0.5 ppm was detected with a response time of 0.83 min; however, severe sensor poisoning occurred. The LOD for CH_3_CN was estimated at 0.6 ppm and for DMMP at 0.04 ppm.

Another application of the Mn_3_O_4_ sensor is described in [75]. In this case, DPGME was detected using Mn_3_O_4_ sensors doped with Au and Ag. The sensor layers were manufactured by depositing Mn_3_O_4_ with the CVD method on the substrate (40 nm grains were obtained) and then introducing metal nanoparticles onto it by radio frequency sputtering. The tests of the sensor’s response to DPGME and other substances (acetone, ethanol, ACN, DMMP) revealed that among the prepared sensors (Au-Mn_3_O_4_, Ag-Mn_3_O_4_ and Mn_3_O_4_), the Au-Mn_3_O_4_ sensor has the highest sensitivity and selectivity towards DPGME. The estimated DPGME detection limit for this sensor was 0.6 ppb (in contrast to 50 ppb for the sensor with undoped Mn_3_O_4_). This effect was attributed to the formation of Schottky junctions in the Au/Mn_3_O_4_ system (in the case of Ag/Mn_3_O_4_, a reduced share of this effect was found due to partial oxidation of Ag(0) to Ag(I) during deposition on Mn_3_O_4_). The formation of metal–semiconductor junctions enabled higher modulation of HAL during interaction with the analyte. The optimum operating temperature of the Au-Mn_3_O_4_ sensor was only 200 °C, while for the other sensors, it was 300 °C. Nevertheless, it was found that during the detection of DPGME, there is a slight poisoning resulting in a prolonged time of complete recovery (although irreversible poisoning did not occur). This effect was attributed to the low operating temperature of the sensor, which reduced the rate of desorption of products formed during DPGME oxidation. Taking into account the high sensitivity, reversibility and selectivity of the Au-Mn_3_O_4_ sensor, it can be concluded that the results presented in this paper are a significant achievement in the field of nitrogen mustard simulant detection. It is also worth noting that the DPGME LOD of 0.6 ppb is many times lower than the IDLH of nitrogen mustard. For this reason, a sensor of this type can potentially be used in devices for the selective detection of DPGME.

In work [73], another manganese oxide was used (MnO_2_), which is an n-type semiconductor. Sensor layers were produced using the plasma enhanced-CVD method, which allowed obtaining elongated MnO_2_ nanoparticles with dimensions of 600 × 90 nm. Then, using radio frequency sputtering, CuO and SnO_2_ dopants were applied. Doping MnO_2_ with other semiconductor oxides was aimed at producing p-n (CuO/MnO_2_) and n-n (SnO_2_/MnO_2_) semiconductor nanojunctions (and, as a result, improving the properties of the sensors resulting from charge transfer processes occurring across the junctions). The SnO_2_/MnO_2_ sensor allowed the detection of DPGME and DMMP with LODs of 0.1 ppm and 2.3 ppb, respectively, at 250 °C. However, the analysis of the results presented in the paper did not allow deeper conclusions about the selectivity of the CuO/MnO_2_ and SnO_2_/MnO_2_ sensors. 

Table 1 presents the most important information regarding some MOS sensor research, the subject of which was the detection of CWAs and their simulants.

## 4. Sensors with Active Layers Using Carbon Nanotubes and Graphene

Carbon nanotubes (CNTs) exhibit extraordinary electrical properties, such as ballistic transport over a few hundred nanometers, high current-carrying capacities (10^8^–10^9^ A/cm^2^) and excellent temperature stability [77]. Because their entire mass is accumulated on the surface, they are characterized by a very large specific surface area (100–1800 m^2^/g) and their electrical properties strongly depend on the environment. These features have made them widely used in gas sensors. CNTs come in the form of single-walled tubes (single-wall CNTs-SWCNTs), which can be metallic or semiconductor, and multi-walled (multi-wall CNTs-MWCNTs), mainly metallic. The planar equivalent of carbon nanotubes is graphene, which has also found wide application in gas sensors.

**Figure 8:**
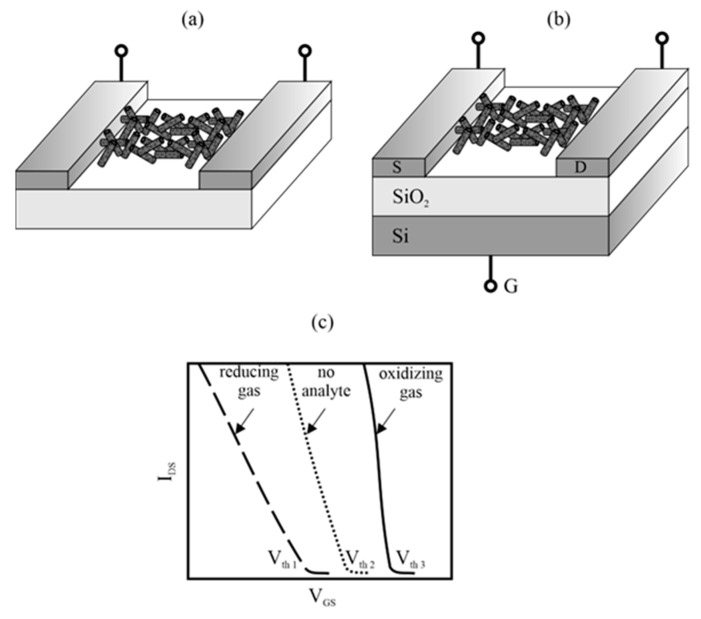
CNT (**a**) chemiresistor, (**b**) field-effect transistor (CNTFET) and (**c**) CNTFET input characteristics after exposure to different analytes.

Sensors with CNTs can work as resistive sensors as well as field effect sensors. The simplest sensor architecture is a chemiresistor, which consists of two electrodes connected by a CNT film (Figure 8a). Resistance variations of the SWCNT network are monitored during gas exposure. At room temperature, the resistance of SWCNT chemiresistors depends on the environment and decreases in an oxidizing atmosphere, while it increases in a reducing atmosphere. Changes in resistance result from charge transfer and changes in the number of holes in nanotubes, which are p-type semiconductors [77]. Chemiresistors show an almost linear response to small concentrations of many different gases. At high concentrations, they tend to become saturated due to the exhaustion of free absorption sites in the active layer.

A typical CNT field-effect transistor architecture is shown in Figure 8b. It consists of CNTs bridging two metallic source-drain electrodes. A doped silicon substrate, separated from the electrodes by a SiO_2_ insulating layer, is used as a gate.

Under constant bias voltage (V_DS_), the conductivity of the semiconducting SWCNTs can be modulated by applying a gate voltage (V_GS_). The device exhibits the characteristics of a p-type transistor in air, as the atmospheric oxygen atoms adsorbed on the semiconductor SWCNTs pull out electrons and create holes. The gate voltage modifies the height of the Schottky barrier built at the junction of the semiconducting SWCNTs electrodes/metal interfaces, and thus the probability of holes passing from the electrodes to the valence band of the semiconducting SWCNTs. The presence of the analyte changes the voltage–current characteristic of the transistor, in particular, the threshold voltage (V_th_). The direction of these changes depends on whether the analyte is an electron donor or acceptor (Figure 8c).

SWCNT field-effect transistors are much more sensitive than MOS sensors and, unlike them, can operate at room temperature. For simple gases (NO_2_, NH_3_), the typical LOD is at the level of a single ppb. The response times of these sensors depend on the structure of the SWCNT layer and are several seconds for single nanotubes. The response time of sensors with thick layers of CNTs is limited by the diffusion rate of the analyte into the bulk of layer, as in the case of oxide sensors. A certain problem is the recovery of SWCNT sensors, as it usually requires many hours of air flushing or a significant increase in temperature, usually to about 200 °C. In FET sensors, this process can be accelerated by changing the polarity of the V_DS_. In this way, a complete recovery was obtained in 200 s [78].

The parameters of the CNT sensor depend on the structure of the CNT film, which in turn depends on the method of preparation and application to the sensor surface. Various methods of depositing CNTs between sensor electrodes are used, the most important of which are CVD [79,80] and drop-casting an SWNT solution onto a sensor substrate [81,82,83,84]. CVD requires high temperatures and specialized equipment. On the other hand, solution-casting techniques are more reproducible, simpler and more efficient, but they usually give weaker electrical contact between CNTs and electrodes [80]. Apart from the simplest techniques of dip-dropping [82,83,85] and spin-coating of the CNT suspension [86,87], dielectrophoresis [88,89] and electrospraying [90] are also used. Often, the substrate is functionalized with amine compounds that adsorb CNTs by electrostatic attraction [88,91].

The main disadvantage of SWCNTs sensors, however, is the lack of selectivity. To solve this problem, many treatments are used, the most important of which is the functionalization of nanotubes [85,87,90], application of a selective diffusion barrier [81], doping with heteroatoms [92,93], decoration by metallic nanoparticles [94] and selection of electrode material [95]. In the case of the detection of organophosphate CWAs, chemical compounds that form hydrogen bonds with them are often used as a coating [84,85].

### 4.1. CNT Sensors

CNTs are a very versatile sensing material, which has also been used in the detection of CWAs and their simulants. So far, sensors with CNT layers have been mainly used for DMMP detection. This compound, like actual CWAs, is a strong electron donor and, as a result, reduces the concentration of holes in the nanotubes that causes an increase in their electrical resistance [78]. This effect is so strong that using CNTs, it is possible to detect DMMP at the sub-ppb level. The second mechanism is the physical adsorption of DMMP on the nanotubes. As a result, DMMP molecules hinder the transport of electrons in nanotubes, which increases their resistance [81,88]. An important feature of nanotube sensors is that they can operate at room temperature. As a result, the energy needed for their operation is lower than in the case of MOS sensors. Significant selectivity towards electron donor substances can be achieved in the presence of non-charge carrier substances, e.g., polyaromatic hydrocarbons and water. Below, we present an overview of CNT sensors used for detecting CWA simulants, mainly DMMP.

Sensors with pristine nanotubes were described in works [78,88,95]. Nowak et al. [78] described two sensors—one with a field-effect transistor and the other with a chemiresistor, in which CNTs were fabricated by CVD. The sensors enabled the detection of DMMP at the ppb level in the presence of some interferents (water, xylene and hexane).

Comparatively good DMMP detection properties were demonstrated by a sensor in which pristine SWCNTs were deposited on the oxidized silicon activated with 3-aminopropyl trimethylsilane [88]. The amino-terminated Si/SiO_2_ wafer surface attracted nanotubes from the solution, creating an SWNTs network on which gold interdigitated electrodes were deposited. As a result, very good electrical contact between the nanotubes and the electrodes was achieved. The properties of the sensor depended on the density of SWCNTs in the sensor layer. A layer containing 30 to 40 tubes per µm^2^ had very good properties. The resistance of such a layer was over 600 Ω. The sensor was characterized by a large change in resistance under the influence of DMMP, fast response, short recovery time and reproducibility. This sensor, like others with nanotube layers, operated at ambient temperature.

Most CNT sensors used for DMMP detection are chemiresistors. However, good detection parameters can also be obtained using field effect sensors, where the metal/CNT Schottky barrier is modulated by polar chemicals. Bondavalli et al. [95] used different electrode materials (Au, Pd, Pt and Ti) with different work functions. As a result, individual sensors were characterized by different sensitivities to DMMP. For Ti/Pd electrodes and V_GS_ = −30 V, V_DS_ = 1 V, the I_DS_ current decreased tenfold after exposure to 1 ppm of DMMP.

In most solutions, nanotubes are covered with a coating material, the task of which is to improve selectivity. Polymers are typically used for this purpose, but not exclusively. In the case of DMMP detection, among others, were used: polyisobutylene [81], polythiophene [87], polyaniline [82,83], DNA [79], ZnO [96], poly[2-methoxy-5-(2-ethyloxy)-p-phenylenevinylene] and poly(methyl methacrylate) [82]. These coatings can be a barrier to interfering agents, and also participate in the detection process themselves. A review of the use of conductive polymers on carbon nanotubes is presented in [97].

In work [81], the cross-linked SWCNTs layer was deposited on polyethylene terephthalate and covered with a 2 µm polyisobutylene film. The polymer isolated the nanotubes from interfering substances such as xylene, hexane and water. As a result, these substances were not detected. However, this layer did not isolate the nanotubes from the nerve agent simulants. In the case of DMMP and DIMP detection, the resistance of the sensor layer increased. With analyte concentrations ranging from 25 to 50 ppm, the change in resistance was 5–8%, with the sensor showing higher sensitivity to DIMP. The authors attributed the better detection of DIMP to the strong interaction of its isopropyl group with nanotubes (DMMP does not have such a group). The concentration-resistance plot was linear for DIMP up to 10,000 ppm and for DMMP up to 5000 ppm. The response time of the sensor was about 20 min, and the recovery, about 10 min. Sensor indications were repeatable.

A selective, repeatable and reproducible DMMP sensor was obtained when SWCNTs were coated with a layer of 4-(hexafluoro-2-hydroxy isopropyl)-functionalized aniline (HFiPA) [84]. SWCNTs were applied to the surface of a polycarbonate membrane with 0.5 μm pores by vacuum filtration. The nanotubes were coated with HFiPA using the drop cast method. The paper compares the detection of DMMP using sensors with both pristine and functionalized CNTs sensors. The HFiPA CNTs sensor showed significantly better performance than the second sensor. For a DMMP concentration of 24 ppm, the response of the HFiPA CNTs sensor was 3.7 times greater than that of the non-functionalized sensor. The response value for the SWCNTs-coated sensor was 16% for 24 ppm and 47% for 1200 ppm DMMP. The functionalized sensor was also characterized by better dynamic properties. Similarly, in the case of the HFiPA CNTs sensor, better selectivity towards DMMP in the presence of benzene, toluene, hexane, dichloromethane, ethanol and water vapor was obtained. The DMMP LOD was 69 ppb. The authors explain these results by the strong interaction of HFiPA acid groups with basic DMMP. The HFiPA has strongly acidic hydrogen atoms in the hydroxyl groups that participate in the formation of hydrogen bonds with organophosphate compounds.

In the work cited above, the selectivity and sensitivity of the sensors were ensured by the HFiP group, which forms hydrogen bonds with organophosphorus compounds. The hexafluorobisphenol moiety works in a similar way. Both groups have been widely used in SWCNTs sensors for DMMP detection [85,87].

In [85], hexafluorobisphenol A was covalently bound to the surface of nanotubes by the reaction of bisphenol hydroxyl groups with carboxyl groups on the surface of SWCNTs. Then the free phenyl groups formed a strong hydrogen bond with the DMMP molecule. As a result, the sensor allowed very good and selective detection of DMMP at sub-ppm concentrations.

Wang [87] studied sensors in which functionalized polythiophene was deposited on SWCNTs. SWCNTs were dispersed in polythiophene substituted with hexafluoroisopropanol (HFIP-PT) or poly(3-hexylthiophene). Sensor layers with a thickness of 50 nm were obtained using the spin-coating technique. Research has revealed that the HFIP-PT sensor performs significantly better than others during DMMP detection. This sensor was able to quickly, reversibly and selectively detect DMMP at the ppb level. The resistance of the sensor layer during the measurements varied from 0.5 to 1.5 MΩ. At the DMMP concentration of 0.05 ppm, the change in conductivity was 1%, and at the concentration of 0.6 ppm, it was 8%. Water did not interfere during measurements because it was detected at a concentration of about 100 times higher than that of DMMP. The mechanism of interaction of DMMP with HFIP-PT consisted of the formation of a hydrogen bond and a change in the conductivity of polythiophene.

The work [98] presents research on a chemiresistor using the composite of SWCNTs and poly(3,4-ethylenedioxythiophene) (PEDOT) polymer functionalized with HFiP groups. The sensor layer was produced by the drop-casting method. The sensor prepared in this way was used to detect DMMP in dry nitrogen and humid air (relative humidity 24%). The obtained DMMP detection limits in dry nitrogen and humid air were 2.7 ppm and 6.5 ppm, respectively. In addition, the sensor showed good selectivity towards DMMP (selectivity was tested in the set of tetrahydrofurane, benzene, ethyl acetate, ACN, CH_3_Cl, hexane, acetone, CH_3_OH, water).

In paper [83], polyaniline was used to cover the SWCNTs network. The SWCNT–polyaniline active layer was placed between the palladium electrodes. DMMP molecules interacting with this layer caused the introduction of electrons, changing the electrical resistance of the layer. In the presence of 1 ppm DMMP, the sensor response was 10.5%. In analogous conditions, the response of the sensor without polyaniline was about 10 times lower. The response of the polyaniline sensor to DMMP was linear over the concentration range of 1 to 5 ppm. The LOD of DMMP was estimated at 1 ppm. The response time of the SWCNT–polyaniline sensor was 5.5 s and was about three times shorter than that of the pristine SWCNT sensor. The sensor operated at room temperature.

The use of polyaniline as a coating for multi-walled carbon nanotubes (MWCNTs) was presented in [82]. In addition to polyaniline, poly[2-methoxy-5-(2-ethyloxy)-p-phenylenevinylene] and poly(methyl methacrylate) were also used. Using the obtained sensors, DMMP and DCM were detected in the presence of other organic compounds. The best results were obtained for MWCNTs coated with polyaniline. The sensor layer, with an initial thickness of 0.8 µm, under the influence of the DMMP, increased its thickness (up to 1.3 µm). In the presence of DMMP, the resistance of the system increased and allowed the detection of DMMP at the ppm level. The detectability of the MWCNT-polyaniline sensor was several times better than that of other sensors. DMMP and DCM were detected in the presence of xylene, tetrahydrofuran, methylethyl ketone, chloroform and dichloromethane.

In addition to polymer coatings, metal oxides have also been used. An example is a sensor with an SWCNT layer covered with ZnO [96]. Using such a sensor, DMMP was detected at the ppm level at room temperature. The thickness of the ZnO layer on the SWCNT surface determined not only the resistance of the sensor, but also the type of conductivity. In the case of thin ZnO layers, the system showed p-type conductivity (resistance of the sensor increased under the influence of DMMP), while for thicker ZnO layers, the layer behaved as an n-type semiconductor.

An unprecedented type of coating was used in [79]. In this case, the authors used single-stranded DNA (ss-DNA) coating to detect DMMP and 2,6-dinitrotoluene (DNT) in the presence of methanol, propionic acid, water and trimethylamine. The ss-DNA/SWCNTs sensor showed significant selectivity towards DMMP and DNT (the signal to interfering agents was from about 5 times (propionic acid) to 500 times lower (trimethylamine), and practically zero in the case of water). At a DMMP concentration of 25 ppm and DNT of 40 ppm, the signal of the device was 14% in both cases. The sensor was characterized by short response and recovery times of a few minutes.

MWCNT functionalized with thiourea groups was used to detect DMMP in [90]. The sensor was made on a textile substrate obtained by the electrospun technique. A polysulfone fiber mat provided flexible, porous staging, which increased the specific surface area of the sensor material. MWCNTs were applied to this substrate using the layer-by-layer assembly technique and then functionalized. The thiourea-functionalized MWCNT sensor was up to three times more sensitive than the sensor with pristine MWCNTs. It is worth noting that the sensor described in [90] is one of the few that can be used to detect DMMP both in water and in air. Its detectability in water was 10 ppb and in air 5 ppb.

### 4.2. Sensors with Graphene and Other Carbon Materials

Graphene is a planar equivalent of CNTs and, as such, is used in resistive and field effect gas sensors [77,86,99,100]. The form of graphene, suitable for sensor applications, is obtained, e.g., by micromechanical exfoliation of graphite [101], CVD [102] and solution-based chemical reduction of graphene oxide-GO [86,99]. Graphene, in its oxidized form, has very low conductivity and is not suitable for sensing applications. Therefore, it is reduced in order to ensure sufficiently high electrical conductivity.

In [99], reduced graphene oxide (RG) was obtained by reducing graphene oxide with p-phenylenediamine and hydrazine. The active layers were applied using the drop drying method, taking advantage of the fact that RG creates dispersions in organic solvents (e.g., in ethanol). In the cited work, DMMP was detected using sensors with graphene obtained by reduction with various reducers. RG obtained as a result of reduction with p-phenylenediamine (RG1) had much better properties than those reduced with hydrazine (RG2). When DMMP was detected at a concentration of 10 ppm, the RG1 sensor had a response 3.3 times greater than that of the RG2. The dependence of the resistance change on the concentration of DMMP in the range from 5 to 80 ppm was linear. The sensor response time was about 18 min, and the recovery time was 6 min (in the case of recovery, IR lamp illumination was used, which significantly accelerated the recovery of the sensor). As the tests revealed, the sensor maintained high repeatability even after several months. In addition, the sensor allowed the selective detection of DMMP in the presence of methanol, dichloromethane, hexane, chloroform and xylene.

The type of chemical compound used for the reduction of graphene oxide has a large impact on the properties of graphene as a sensor material [100]. In the cited work, the influence of the graphene reduction method on the properties of the DMMP sensor was investigated. Graphene oxide was reduced with hydrazine hydrate, ascorbic acid and sodium borohydride. In the case of ascorbic acid and sodium borohydride, the sensors were much more sensitive than in the case of reduction by hydrazine. The reason for this was the presence of nitrogen atoms in hydrazine-reduced graphene. The presence of these atoms adversely affected the interaction with DMMP and changes in the conductivity of the graphene layer. On the basis of the manufactured sensors, a sensor array was then built. The sensor array made it possible to distinguish DMMP from other substances such as ethanol, methanol, n-hexane, acetonitrile, diethyl ether, dichloromethane, chloroform and acetone. Principal component analysis was used to interpret the array signal. The detectability of DMMP was adversely affected by moisture in the analyzed gas.

In [77], the properties of three graphene materials were compared using ab into computational methods, with particular emphasis on the mechanisms of interaction with DMMP and their molecular structures. The subject of the study was pristine graphene, boron nitride graphene (BN-graphene) and aluminum nitride graphene (AlN-graphene). The energies of these interactions, including adsorption energies and charge transfer, were determined by simulation. A particularly strong interaction occurred between DMMP and AlN-graphene. Adsorbing DMMP molecules on the surface of this graphene caused the semiconductor graphene to acquire metallic properties. This is a property that favors very good DMMP detection.

In a paper published in 2021, graphene in the form of quantum dots (GQD) combined by a strong π-π bond with cobalt phthalocyanine (PC) derivatives was used [103]. Graphene quantum dots increased the conductivity of phthalocyanine. The optimal weight ratio of PC to GQD was 9:1. The device with such a sensor layer enabled the detection of DMMP at a concentration of 500 ppb in a short time at room temperature. The selectivity, reproducibility and stability of the sensor were very good. However, the sensor was characterized by an extended recovery time. A laser or IR lamp was used to achieve reversibility.

Graphene analogs are two-dimensional nanomaterials obtained from elements of group IVA and VA. One of them is ε-arsenene, which is a nanosheet of the allotrope of arsene. It was used in a sensor for detecting sulfur mustard simulant-2-chloroethyl dichloromethyl sulfide (CECMS) [104]. Using the density functional theory method, the mechanism of interaction of HD with ε-arsenene was investigated. The presence of the CECMS was recorded by the current flowing through the sensor. The value of the current, for fixed CECMS concentrations depended on the voltage applied to the sensor electrodes in a highly non-linear way. The sensor was able to selectively detect CECMS against moisture, CO and CO_2_.

One type of sensor layer is materials in which semi-conductive organic polymers are mixed with carbon black [105]. The mixtures are applied to glass substrates, which are then fitted with gold electrodes. In the cited work, 10 such sensors with different polymers were prepared. These sensors were combined into an array, and the detection capabilities of DMMP and DIMP were investigated. Sarin simulants have been detected in the presence of water vapor, methanol, benzene, toluene, diesel fuel, tetrahydrofuran and others. Principal component analysis was used to differentiate the analytes. DMMP and DIMP were distinguishable from all other substances. DMMP was detected at 9 ppb and DIMP at 110 ppb. The detection of individual substances was related to their solubility in polymers.

In [106], a flexible sensor layer was obtained by covering the carbon nanofiber sheet (CNS) with MnO_2_. The sensor obtained in this way was characterized by high mechanical durability. It was used to detect DMMP. Both MnO_2_ and CNS participated in the detection process according to the charge-transfer mechanism, causing an increase in the resistance of the sensor layer. The detectability of the MnO_2_/CNS sensor was clearly better than the CNS sensor used here as a reference. In the first case, DMMP could be detected at 0.1 ppb, and in the second at 0.1 ppm. The sensor showed good stability and reversibility. At the same time, the MnO_2_/CNS sensor showed high selectivity to DMMP in the presence of acetone, benzene, chloroform, dimethylformamide, ethanol, hexane and toluene.

Table 2 summarizes the most important features of CNTs sensors that were used for DMMP detection.

Significant progress in the technology and availability of carbon nanostructures and 2D materials has enabled dynamic development in the field of non-oxide semiconductor sensors. In contrast to MOS-type sensors, these materials offer much wider possibilities of tailoring selectivity. Especially the use of functionalizing carbon materials with specific polymers gives good results and it seems that thanks to these materials, it will be possible to build ready-to-use sensors in the near future.

In the case of non-oxide sensors, it is also very important that they usually work at lower temperatures than MOS sensors, which translates into lower electricity consumption. For a single sensor, power consumption of 100–300 mW (typical power consumption of thick-film MOS sensors, most of which is due to heating the sensor to a temperature of several hundred Celsius degrees) is acceptable even for portable devices. However, in the case of a sensor array composed of several or a dozen or so sensors, the total energy consumption can be a big problem. This issue is all the more important because, so far, a very effective way to improve the selectivity of devices based on semiconductor sensors has been the fabrication of arrays.

Despite the apparent advantages, many issues related to non-oxide sensors remain to be resolved. Of particular importance is the improvement of recovery time. In this case, methods involving the use of IR lamps, a laser or reverse polarity in the case of FET transistors seem very promising and may result in implementations in ready-to-use devices in the future.

## 5. Sensors with Other Types of Active Layers

Organic semiconductors, e.g., in the form of polymers, have also been used as active materials in semiconductor sensors [107,108]. Semiconductor polymers are suitable materials for such sensors for a number of reasons. However, due to their dielectric and electrostatic properties, there are some problems with their bonding to metal electrodes.

In the literature, one can find sensors with active layers made of phthalocyanines [109,110]. In [110], sensor films consisting of layers of palladium and cobalt phthalocyanine (Co-Pc), as well as palladium and metal-free phthalocyanine (H_2_-Pc), were obtained. The layers were prepared by depositing 100 nm phthalocyanine and 10 nm palladium on the substrate. DMMP and several other organic compounds have been detected using such sensors. Under the influence of DMMP, the primary resistance of the sensor decreased. As the research showed, the sensor with H_2_-Pc was characterized by better DMMP detection than the sensor with Co-Pc. Methanol, ethanol, iso-propanol and water vapor did not interfere with DMMP detection at 10 to 60 ppb in the H_2_-Pc sensor and 10 to 300 ppb in the Co-Pc sensor.

Several different phthalocyanines were also studied in [109]. Thin layers of phthalocyanine, 50 nm thick, were deposited on a silicon substrate covered with a 1 µm layer of SiO_2_. The following phthalocyanines were used as semiconductor sensor layers: metal-free, cobalt, nickel, copper and zinc. Using the produced sensors, DMMP and eleven other chemical compounds from the group of Lewis bases and forming hydrogen bonds with phthalocyanines were detected. The detection of individual substances was related to the enthalpy of the analyte–phthalocyanine interaction. The enthalpy value also affected the recovery time. In the case of metal phthalocyanines, the mechanism of interaction of the analytes with them was related to the Lewis basicity. The interaction of the analytes with metal-free phthalocyanine was associated with the formation of hydrogen bonds.

## 6. Conclusions

Semiconductor sensors are a diverse group of devices whose common feature is the semiconductor material used as a receptor. It has been 61 years since the construction of the first semiconductor gas sensor [15], and the first paper on their application for the detection of CWA simulators was published 30 years ago [50].

As shown in this review, many papers have been published over the years on the use of semiconductor sensors in the detection of CWAs and their simulants. In some of them, systems characterized by very good sensor properties, such as the detection of toxic substance simulants at the level of single ppb (i.e., concentrations much lower than the IDLH of actual CWAs [61]), high speed of action or selectivity. The fact is, however, that at the moment, there are no CWA detection devices on the market based only on semiconductor sensors (although there are ready-made solutions, or at the stage of an advanced prototype, in which these sensors are used as an auxiliary technique [111,112]).

The analysis of the studies presented in this review indicates that the selectivity of the sensors is still a big problem. The methods used to improve selectivity, especially in the case of MOS sensors, are insufficient, and in most studies, they are based on an empirical approach and not on the principles resulting from the analysis of the sensor operation mechanism. Significantly more advances in improving selectivity have been made with sensors using non-oxide semiconductors, which often rely on more selective interactions with the analyte (e.g., solvation interactions) than with MOS sensors. The approach involving the construction of sensor arrays also seems promising.

In conclusion, despite significant achievements in this field, the CWA detection technique based solely on semiconductor sensors is rather far from being implemented in commercial devices. A number of unsolved problems, the biggest of which is poor selectivity, have meant that, despite many years of research, it has not been possible to build ready-made devices so far. On the other hand, sensors using oxide-free semiconductors have great potential. In this case, the possibilities of obtaining highly selective sensors are much greater than for MOS sensors. In addition, these sensors typically operate at room temperature, unlike MOS sensors, which typically operate at temperatures of 300–500 °C.

In our opinion, further development of semiconductor sensor technology will focus on sensors with non-oxide semiconductors (in particular, on sensors with layers using CNTs and graphene). This development will be possible thanks to the increasingly better mastered technology and the improving availability of carbon nanomaterials used for their construction.

## Figures and Tables

**Table 1:** MOS sensors used for detection of CWAs and their simulants.

No.	Basic Metal Oxide Semiconductor	Sensitizers	Analytes	Value of Response to the Most Detectable Analytes *	Temperature [°C]	Limit of Detection (LOD)	Response/Recovery Time to the Most Detectable Analytes	Ref.
1	SnO_2_ NPs 40 nm/polycrystalline thick film	none	DMMP, ACN, DCM, DPGME	DMMP: 60% at 0.5 ppm	350	n/a	minutes/irreversible	[43]
2	SnO_2_ NPs/polycrystalline thick film	NiO, MoO_3_, Sb_2_O_3_	DMMP	DMMP: 70% at 0.5 ppm	350	n/a	minutes/full recovery in about 100 min	[51]
3	SnO_2_ NPs 10 nm/polycrystalline thick film	none	DMMP	DMMP: 7900% at 5 ppm	500	n/a	tens of minutes/tens of minutes, partial recovery	[58]
4	SnO_2_ nanospheres/thin film	aging with ethanol	Sarin (GB), DMMP	GB: approx. 30% at 47 ppb	300	6 ppb	5 min/5 min	[35]
5	SnO_2_ nanowires (lateral dimension 100 nm)/thin film	none	DMMP	DMMP: 170% at 0.2 ppm	500	n/a	tens of seconds/minutes, poisoning	[57]
6	SnO_2_ single, monocrystalline nanobelt	none	DMMP	DMMP: 5% at 78 ppb	500	n/a	seconds/tens of seconds	[64]
7	SnO_2_/polycrystalline thick film	oxygen vacancy enrichment/Au	DMMP	DMMP: 19% at 240 ppb	320	4.8 ppb	26 s/32 s, full recovery	[66]
8	ZnO NPs 25 nm	Al.	2-CEES	2-CEES: 95,420% at 20 ppm	500	n/a	seconds/minutes	[46]
9	ZnO NPs 25 nm	Al.	DMMP	DMMP: 434,700% at 10 ppm	350	100 ppb	2 s/96 s, full recovery	[70]
10	ZnO “nanoflowers” with a height of several μm	CuO	DMMP	DMMP: 62,621% at 10 ppm	350	n/a	26 s/ten of minutes, poisoning	[40]
11	In_2_O_3_ NPs 21 nm/polycrystalline thick film	none	Sarin, soman, sulfur mustard (HD), simulants…	GB: 216% at 1 ppm; HD: 347% at 1 ppm	400	GB: 10 ppb; HD: 10 ppb	GB: 1 min/several minutes, poisoning; HD: 1 min/3 min, full recovery	[34]
12	CdSnO_3_ NPs 5 nm/polycrystalline thin film	Pt	2-CEES, CEPS, DMMP	2-CEES: 5863% at 4 ppm	250	n/a	seconds/minutes	[28]
13	Mn_3_O_4_ NPs 80 nm/polycrystalline thin film	none	DMMP, CH_3_CN	DMMP: 24% at 0.5 ppm	200	40 ppb	50 s/tens of minutes, very significant poisoning	[41]
14	Mn_3_O_4_ NPs/polycrystalline thin film	Au	DPGME, DMMP, ACN	DPGME: approx. 280% at 0.5 ppm	200	0.6 ppb	minutes/tens of minutes, slight poisoning	[76]
15	MnO_2_ elongated grain 90 × 600 nm/polycrystalline thin film	SnO_2_	DPGME, DMMP	DPGME: approx. 2% at 0.5 ppm; DMMP: approx. 5% at 0.5 ppm	250	DPGME: 0.1 ppm; DMMP: 2.3 ppb	minutes/tens of minutes (poisoning in case of DMMP)	[73]

* all response values expressed as percentage according to equation: |R_a_−R_g_|/R_a_ × 100%. Values were recalculated to this form on the basis of R_a_ (G_a_) and R_g_ (G_g_) reported in literature.

**Table 2:** Sensors with carbon receptors used for DMMP detection.

No.	Type of Transducer	Method of CNTs Deposition	Coating/Bonding	Response	Limit of Detection (LOD)	Response/Recovery Time	Ref.
1	FET/SWCNT	CVD on SiO_2_	none	n/a	sub ppb	1500 s for 1 ppb/by appl. positive bias to FET gate-200 s	[78]
2	resistor/SWCNT	CVD on quartz	none	1.5 at 1 ppb	sub ppb	n/a	[78]
3	resistor/SWCNT	from solution on the charged surface of activated silicon	none	2 at 10 ppm	n/a	tens of minutes/5 min	[88]
4	FET/SWCNT	spin-coating	none	90% at 1 ppm (derived from change in current I_DS_)	approx. 10 ppb	n/a	[95]
5	resistor/SWCNT	vacuum filtration + drop cast	4-(hexafluoro-2-hydroxy isopropyl)aniline/non-covalent	16% at 24 ppm	69 ppb	192 s/90 s	[84]
6	resistor/SWCNT	dip-dropping	polyisobutylene/non-covalent	<5% at 20 ppm	n/a	1200 s/600 s	[81]
7	resistor/SWCNT	immersion	none	3.6% at 1 ppm	0.15 ppm	18 min/12 min with IR lamp	[91]
8	resistor/SWCNT	dip-dropping	hexafluorobisphenol A/covalent	5.1% at 20 ppm	<0.5 ppm	960 s/720 s	[85]
9	resistor/SWCNT	spin-coating	hexafluoroisoproanol functionalized polythiophene	1% at 0.05 ppm	1 ppb	n/a	[87]
10	resistor/SWCNT	dip-dropping	polyaniline/non-covalent	10.51% at 1 ppm	n/a	5.5 s	[83]
11	resistor/MWCNT	dip-dropping	polyaniline/non-covalent	1300% at 797 ppm	n/a	n/a	[82]
12	resistor/SWCNT	spin coating (CNTs) end magnetron sputtering (ZnO)	ZnO	approx. 4% at 5 ppm	<0.5 ppm	approx. 5 min/20 min	[96]
13	FET/SWCNT	catalytic CVD on SiO_2_/Si, dip-dropping DNA	single-stranded-DNA	14% at 25 ppm	n/a	minutes	[79]
14	resistor/MWCNT	layer-by-layer assembly	thiourea/covalently	0.3% at 10 ppb	5 ppb—in air; 10 ppb—in water	minutes	[90]
15	resistor/graphene	dip-dropping	none	5% at 5 ppm	<1 ppm	1080 s/360 s	[99]
16	resistor/graphene	dip-dropping	none	40% at 20 ppm	<1 ppm	10 min/n/a	[100]
17	resistor/graphene	dip-dropping	Co phtalocyanine + heksafluorisopropanol or hexafluorbisphenol A	9% at 20 ppm	<1 ppm	600 s/630 s with laser	[103]
18	resistor/carbon nanofiber	carbonization of polyacrylonitrile nanofibers sheet soaked in polypyrrole and KMnO_4_	MnO_2_	4% at 0.1 ppb	0.1 ppb	seconds	[106]

## Data Availability

The data presented in this study are available on request from the corresponding author.
